# Faba Bean Cultivation – Revealing Novel Managing Practices for More Sustainable and Competitive European Cropping Systems

**DOI:** 10.3389/fpls.2018.01115

**Published:** 2018-08-02

**Authors:** Anestis Karkanis, Georgia Ntatsi, Liga Lepse, Juan A. Fernández, Ingunn M. Vågen, Boris Rewald, Ina Alsiņa, Arta Kronberga, Astrit Balliu, Margit Olle, Gernot Bodner, Laila Dubova, Eduardo Rosa, Dimitrios Savvas

**Affiliations:** ^1^Department of Agriculture, Crop Production and Rural Environment, University of Thessaly, Volos, Greece; ^2^Laboratory of Vegetable Production, Department of Crop Science, Agricultural University of Athens, Athens, Greece; ^3^Institute of Plant Breeding and Genetic Resources ELGO-DEMETER, Thessaloniki, Greece; ^4^Pūre Horticultural Research Centre, Pūre, Latvia; ^5^Institute of Horticulture, Latvia University of Agriculture, Jelgava, Latvia; ^6^Department of Horticulture, Technical University of Cartagena, Cartagena, Spain; ^7^Department of Horticulture, Division of Food Production and Society, Norwegian Institute of Bioeconomy Research (NIBIO), Oslo, Norway; ^8^Department of Forest and Soil Sciences, University of Natural Resources and Life Sciences, Vienna, Austria; ^9^Institute of Soil and Plant Sciences, Latvia University of Agriculture, Jelgava, Latvia; ^10^Department of Plant Breeding and Genetics, Institute of Agricultural Resources and Economics, Priekuli, Latvia; ^11^Department of Horticulture and Landscape Architecture, Agricultural University of Tirana, Tirana, Albania; ^12^Estonian Crop Research Institute, Jõgeva, Estonia; ^13^Department of Crop Sciences, University of Natural Resources and Life Sciences, Vienna, Austria; ^14^UTAD-CITAB – Centre for the Research and Technology of Agro-Environmental and Biological Sciences, University of Trás-os-Montes and Alto Douro, Vila Real, Portugal

**Keywords:** landraces, legume, nutritional value, soil fertility, sustainability, weed management, *Vicia faba*

## Abstract

Faba beans are highly nutritious because of their high protein content: they are a good source of mineral nutrients, vitamins, and numerous bioactive compounds. Equally important is the contribution of faba bean in maintaining the sustainability of agricultural systems, as it is highly efficient in the symbiotic fixation of atmospheric nitrogen. This article provides an overview of factors influencing faba bean yield and quality, and addresses the main biotic and abiotic constraints. It also reviews the factors relating to the availability of genetic material and the agronomic features of faba bean production that contribute to high yield and the improvement of European cropping systems. Emphasis is to the importance of using new high-yielding cultivars that are characterized by a high protein content, low antinutritional compound content, and resistance to biotic and abiotic stresses. New cultivars should combine several of these characteristics if an increased and more stable production of faba bean in specific agroecological zones is to be achieved. Considering that climate change is also gradually affecting many European regions, it is imperative to breed elite cultivars that feature a higher abiotic–biotic stress resistance and nutritional value than currently used cultivars. Improved agronomical practices for faba bean crops, such as crop establishment and plant density, fertilization and irrigation regime, weed, pest and disease management, harvesting time, and harvesting practices are also addressed, since they play a crucial role in both the production and quality of faba bean.

## Introduction

Faba bean (*Vicia faba* L.) is one of the most globally important legume crops. Its global acreage declined from 3.7 to 2.1 million ha between 1980 and 2014, and yields are highly variable within specific countries (FAO, 2017). Despite the decreasing acreage, however, productivity per area has tended to increase, due to a reduced susceptibility to abiotic and biotic stresses (Link et al., 2010; Sillero et al., 2010; Singh et al., 2012). The global production of faba bean grain in 2014 was 4.1 million tons, which is approximately 21% greater than in 1994 (FAO, 2017). The fresh and dry seeds of faba bean are used for human consumption; they are highly nutritious because they have a high protein content (up to 35% in dry seeds), and are a good source of many nutrients, such as K, Ca, Mg, Fe, and Zn (Lizarazo et al., 2015; Longobardi et al., 2015; Neme et al., 2015). Faba bean seeds also contain several other bioactive compounds, such as polyphenols (Turco et al., 2016), carotenoids (Neme et al., 2015), and carbohydrates (Landry et al., 2016). However, the chemical composition is strongly influenced by variety, as well as environmental and management conditions (Mona et al., 2011; Cazzato et al., 2012; Witten et al., 2015).

The inclusion of faba bean in cropping systems improves soil fertility. Its high efficiency in establishing symbiosis with specific *Rhizobium* bacteria, and the concomitant biological nitrogen fixation (BNF), is associated with a reduced need for fertilizer input in arable lands and increased soil biological activity. The two main agricultural practices that benefit from BNF are crop rotation cycles that include legumes, and the intercropping of legumes with crops that are incapable of fixing N, such as cereals or horticultural crops (Jensen et al., 2010). The amount of nitrogen that can be fixed by faba bean is mainly dependent on the cultivar, local farming practices (such as nitrogen and phosphorus fertilization), soil properties, and the presence of symbiotically effective rhizobia in the soil (Schubert et al., 1990; Adak and Kibritci, 2016; Argaw and Mnalku, 2017).

Several studies have shown that incorporating legume residues into the soil as green manure increases growth and yield in many crops, such as canola, maize, potato, and wheat (Sincik et al., 2008; Miller et al., 2011; Bilalis et al., 2012; O’Donovan et al., 2014). According to Neugschwandtner et al. (2015), faba bean can fix up to 200 kg N ha^-1^, while the incorporation of legume residues into the soil improves soil properties such as organic matter content, bulk density, porosity, and field capacity (Mandal et al., 2003; Salahin et al., 2013; Adekiya et al., 2017). However, in spite of the multiple advantages of using faba bean in crop rotation systems, the area used for its cultivation has decreased in most European countries in comparison with the 1981–1990 period, as reported previously (FAO, 2017).

Given the importance of faba bean in agroecosystems, this review aims to provide an up-to-date overview of the genetic material, its nutritional value, cropping practices, and the role of this legume species in maintaining sustainability in agricultural production in Europe. Cultivation practices that could improve the production and nutritional value of faba bean are highlighted; the main constraints in its commercial production and an overview of its adaptability to different abiotic stresses are also presented. The importance of screening for and creating new varieties with an increased resilience to biotic and/or abiotic stress is emphasized.

## History, Origin, And Distribution

The genus *Vicia* L. belongs to the family Fabaceae. Knowledge of the wild progenitor and area of origin of the genus, and subsequent steps in the domestication of its most important member species, *V. faba* L., is scarce and disputed (Shiran et al., 2014). The Near East is considered a center of origin for faba bean (Cubero, 1974), while China seems to be a secondary center of faba bean genetic diversity (Zong et al., 2009, 2010). In support of Cubero’s findings, Caracuta et al. (2016) have identified seeds of a potential ancestor of faba bean adjacent to Mount Carmel, Israel – the remains were C-dated to 14,000 years BP (before present). Moreover, Caracuta et al. (2015) have determined that faba bean was already domesticated about 10,200 years BP in the Lower Galilee, Israel. In any case, faba bean can be considered one of the earliest domesticated crops in light of numerous archeological findings in Eurasia and Africa which date back to the early Neolithic (Duc et al., 2015a).

*Vicia faba* has a large genetic diversity. According to Duc et al. (2010, 2015a), >38,000 accessions of faba bean germplasm are conserved globally in numerous gene banks, as well as at the International Center for Agricultural Research in Dry Areas (ICARDA). Research conducted by the EUROLEGUME consortium has shown that potentially many more genotypes are available locally in Europe, at farms and in breeders’ collections (Lepse et al., 2017); a small selection of faba bean genotypes of European origin are shown in **Table 1**. The genetic diversity of *V. faba* accessions has been assessed in various studies and marker systems (Zeid et al., 2003; Zong et al., 2009; Oliveira et al., 2016; Sallam et al., 2016; Göl et al., 2017). In practice, however, a continuous variation in most morphological, (eco-)physiological, and chemical traits has been observed, making it challenging to achieve a discrete differentiation between varieties. A study by Martos-Fuentes (2017) shows that NGS (next-generation sequencing)-based genotyping of faba bean using multiple barcoded samples is a feasible method. The author’s resulting distance matrix shows that some *V. faba* clades were exclusively formed by accessions from a specific country, while others were interspersed, indicating that genetic and geographic distances do not always correspond.

**Table 1:** Selected *Vicia faba* var. *minor* and var. *major* genotypes originating from various European countries.

Country of origin	Genotypes	Reference
Albania	BG 144001, BG 148005, BG 146003, BG 145002, BG 793710, BG 788700, BG 789701, BG 787699, BG 790705, BG 144001, BG 147004, AUT 0001, and AUT 0002	Nasto et al., 2016
Austria	Di-2384, Di-2385, and Di-2310	Moussart et al., 2008
Belgium	V-211	Rubiales et al., 2014
Bulgaria	V-294	Rubiales et al., 2014
Denmark	Di-2387	Moussart et al., 2008
France	Di-279, Di-1626, Di-276, Di-277, and Di-281	Moussart et al., 2008
Germany	Di-2436	Moussart et al., 2008
Greece	Ftakoukia, Platokoukia, Stenokoukia, AUAANDROSfb001, AUALEFKADAfb001, and AUAMANIfb001	Thomas et al., 2013; Ntatsi et al., 2018
Hungary	V-319	Rubiales et al., 2014
Netherlands	Di-1216	Moussart et al., 2008
Latvia	Bauska, Priekulu 32, Priekuïu vietejas, Valmiera, Džūkstes, Zaigas, Puntuïa tumŠās, Cēres, Puntuïa gaiŠās, Iras, VF_01, VF_02, and V-329	Rubiales et al., 2014; Dane et al., 2017; Bodner et al., 2018
Russia	V-1271	Rubiales et al., 2014
Spain	Di-1653, V-903, and V-1196	Moussart et al., 2008; Rubiales et al., 2014
Sweden	Gubbestad	Bodner et al., 2018
Ukraine	V-268	Rubiales et al., 2014

Evolution of the species was accompanied by intensified cultivation, with selection for different traits. The genotypes of *V. faba* are commonly classified into three main botanical varieties according to seed size: (a) *V. faba* var. *major* with large seeds, (b) *V. faba* var. *minor* with small seeds, and (c) *V. faba* var. *equina* with medium seeds (Cubero, 1974; Crépon et al., 2010; Pietrzak et al., 2016), the first two of which are relevant in European agriculture. However, faba bean germplasm is also grouped into spring and winter types, according to frost tolerance, delimiting target climatic zone, and sowing time, and according to the ability to adapt to oceanic or continental (i.e., drought-prone) climates (Moreno and Martinez, 1980; Link et al., 2010; Flores et al., 2013). Recently, Zhao et al. (2018) have shown that cultivar groups featuring differential root system architectures exist independently of botanical variety within Europe, but that, for example, cultivars from Portugal possess greater and coarser but less frequent lateral roots at the top of the taproot in comparison with Northern European cultivars, potentially enhancing water uptake from deeper soil horizons.

## Morphological Description and Botanical Characterization

Faba bean is a cool season annual legume (Bilalis et al., 2003) that forms coarse, upright, hollow, and unbranched stem(s) from the base, and grows between 0.1 and 2 m tall (Bond et al., 1985; Duc et al., 2015a; Heuzé et al., 2016). Stem growth is indeterminate, and some cultivars are prone to lodging. The leaves are alternate, pinnate, and consist of two to six leaflets, which are up to 8 cm long without tendrils (Bond et al., 1985). The flowers have a typically papilionaceous structure and are grouped in inflorescences; they are either pure white in color or with diffuse anthocyanin pigmentation on all petals, while black spots are often present on the wing petals (Bond et al., 1985; Duc et al., 2015a; Heuzé et al., 2016). Seeds, which vary considerably in size, are oblong to broadly oval with a prominent hilum at the end; their color can be yellow, green, brown, black, or violet, and sometimes seeds are spotted (Nozzolillo et al., 1989; McDonald and Copeland, 1997; Duc et al., 2015a). Faba bean plants feature a robust taproot with frequent lateral root branching from the top of the tap root; nitrogen-fixing nodules containing rhizobia occur on both the tap and lateral roots (Bond et al., 1985). Root traits of European accessions vary profoundly (Zhao et al., 2018), and are largely influenced by the tillage regime (Muñoz-Romero et al., 2011).

Faba bean is generally considered day-neutral, while some accessions require long-day conditions in order to flower. However, thermal time is the most important contributor to flowering progress in faba bean, with approximately 830–1000°days above 0°C being required; winter faba bean genotypes require vernalization (Patrick and Stoddard, 2010). For northern European cropping systems, Bodner et al. (2018) have recently reported results of 650°days and 0°C base temperature before flowering; this potentially reflects a photoperiodic sensitivity toward long-day conditions in faba bean. In a recent study, Cao et al. (2017) found that several potential regulators are implicated in the vernalization process in faba bean. Faba bean is a self-pollinated plant with significant levels of cross-pollination (Suso et al., 1996; Chen, 2009). The main pollinating insects are honeybees (*Apis* spp.) and bumblebees (*Bombus* sp.); the benefits of insect pollination for yield have been well documented (Stoddard, 1991; Cunningham and Le Feuvre, 2013; Bishop et al., 2016).

## Adaptability of Faba Bean to Abiotic Stress

**FIGURE 1:**
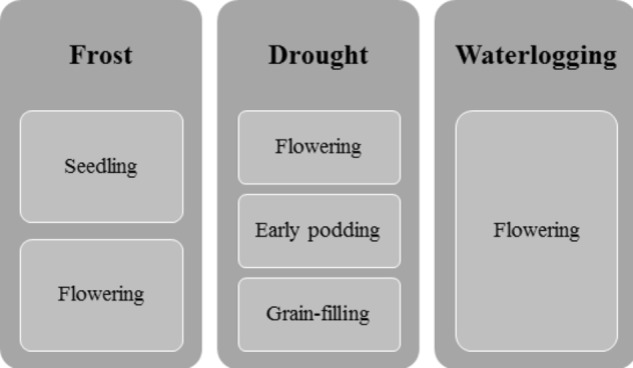
Critical stages of faba bean growth in responding to the main abiotic stress factors.

Drought and heat are considered major constraints in faba bean growth and production in Europe. The most drought-sensitive growth stages are flowering, early podding, and grain filling (**Figure 1**; Mwanamwenge et al., 1999; Katerji et al., 2011). However, faba bean varieties differ widely in drought tolerance (Girma and Haile, 2014). One of the mechanisms apparent in drought-tolerant varieties or genotypes is proline accumulation (Migdadi et al., 2016; Abid et al., 2017), but a differential root architecture, influencing access to water, which differentiates varieties originating from Northern vs. Southern Europe, has also recently been identified (Zhao et al., 2018).

According to Maqbool et al. (2010), faba bean is susceptible to frost during its reproductive stages. Recent studies, however, have identified some frost-tolerant genotypes, which could be used in breeding programs (Stoddard et al., 2006; Sallam et al., 2015). Hardening seedlings through exposure to low non-freezing temperatures before the onset of winter may enhance plant tolerance to frost (Arbaoui and Link, 2008).

Waterlogging, e.g., during flowering, limits faba bean growth and yield (Pampana et al., 2016). The negative effects of waterlogging on growth and other physiological traits (i.e., chlorophyll a and b) persist even after cessation of soil flooding (Pociecha et al., 2008). However, faba bean is considered the most tolerant to waterlogging of the cool-season grain legumes (Solaiman et al., 2007).

Excess soil salinity affects both growth and nitrogen fixation in faba bean plants, which are considered moderately tolerant of soil salinity (Bulut et al., 2011). Katerji et al. (2011) report a yield reduction in faba bean at soil salinity levels of ≥6.5 dS m^-1^. According to del Pilar Cordovilla et al. (1999), root systems are more sensitive than shoots to salinity. In addition, at high salinity levels, nitrogenase activity and nodulation are suppressed (Abd-Alla et al., 2001). These researchers observe that high salinity treatments of 80 and 120 mM NaCl caused a significant reduction in faba bean shoot biomass of 25 and 49%, respectively. Moreover, nitrogen accumulation in the shoots reduced by 36 and 63% at salinity levels of 80 and 120 mM NaCl, respectively (Abd-Alla et al., 2001). Measures undertaken to ameliorate the negative effects of salinity on faba bean plants include foliar application of silicon or inoculation with *Pseudomonas fluorescens* (Hellal et al., 2012; Metwali et al., 2015). Salinity-tolerant faba bean genotypes are also available; one example is the line “VF112,” which has been reported as salt-tolerant because salt stress had no effect on its growth or nitrogen fixation (del Pilar Cordovilla et al., 1995). Examples of genotypes with enhanced tolerance to abiotic stresses are presented in **Table 2**.

**Table 2:** Responses of different *Vicia faba* genotypes to abiotic and biotic stresses.

Genotypes	Type of abiotic stress	Level	Reference
Boxer	Heat stress	T	Zhou et al., 2018
FAB6600	Cold stress	S	Zhou et al., 2018
NGB8639	Cold stress	T	Zhou et al., 2018
FAB7024	Cold stress	T	Zhou et al., 2018
S_145, S_004, S_081, S_151, S_299	Frost stress	T	Sallam et al., 2017
S_165, S_129, S_232, S_235, S_111	Frost stress	S	Sallam et al., 2017
C5	Drought stress	T	Siddiqui et al., 2015
Zafar 1	Drought stress	T	Siddiqui et al., 2015
C4, G853	Drought stress	S	Siddiqui et al., 2015
CS20-DK and NC-58	Drought stress	T	Girma and Haile, 2014
Giza 3	Drought stress	S	Abid et al., 2017
Hara	Drought stress	T	Abid et al., 2017
Fiesta VF, Acc 1487/7, Acc 1512/2	Salt stress	T	Tavakkoli et al., 2012
VF46, VF64, and VF112	Salt stress	T	del Pilar Cordovilla et al., 1995
Baraca	*Orobanche crenata*	T	Rubiales et al., 2014
V-26, V-255, V-958, V-1020, V-1085, V-1117, and L-831818	*Ascochyta fabae*	T	Rubiales et al., 2012
BPL 710, ILB 4726, ILB 5284, 132-1, 135-1, 174-1	*Botrytis fabae*	T	Villegas-Fernández et al., 2012

*T, Tolerant; S, sensitive*.

Nitrogen fixation and growth in faba bean are adversely affected by low soil pH (Schubert et al., 1990; Belachew and Stoddard, 2017). Faba bean grows best in soils with a pH ranging from 6.5 to 9.0 (Jensen et al., 2010). At low pH levels (<5.4), BNF is significantly lower than at higher pH levels (>6.2); at pH levels of <4.7, plants show N deficiency symptoms during the early growth stages (Schubert et al., 1990).

## Agronomy

### Crop Sowing and Rotation

Faba bean is usually planted in the autumn, in areas of Europe characterized by mild winter climatic conditions (Bilalis et al., 2003). In cooler agroclimatic zones, sowing is postponed until the end of winter or early spring to prevent frost damage (Sallam et al., 2015). In some areas of the Mediterranean Basin, the earliest varieties can be sown at the end of summer, with the aim of harvesting them by the end of autumn (Cubero, 2017).

The main tillage operations during the sowing period include moldboard plowing (20–40 cm depth) and harrowing, followed by light duty plowing, the last of which is commonly performed using a rotary tiller. Several studies also show that reduced tillage and no-tillage are viable alternatives to conventional tillage in faba bean crops (López-Bellido et al., 2003; Lestingi et al., 2011; Muñoz-Romero et al., 2011; Giambalvo et al., 2012).

Faba bean is usually sown in rows 10–30 cm apart (Bozoğlu et al., 2002; Yucel, 2013), using either a spacing drill (placing 2–3 seeds per hole) or seed drill. The required seed amount ranges between 70 and 200 kg ha^-1^, dependent on seed size and planting density. According to Siddique and Loss (1999), the recommended sowing depth is 5–8 cm. Germination takes place in 4–12 days, and the optimum temperature for germination is 20°C (Khamassi et al., 2013a).

The key agronomic and economic advantage provided by faba bean and other legumes in crop rotation is BNF (Köpke and Nemecek, 2010). The N benefit provided for subsequent crops is often high; a review by Jensen et al. (2010) has demonstrated substantial savings (up to 100–200 kg N ha^-1^) in the amount of N fertilizer required for subsequent crops. Thus, the inclusion of faba bean in crop rotation reduces the need for inorganic N fertilizer, and consequently reduces CO_2_ emissions (Jensen et al., 2012). Other benefits provided by faba bean in rotation systems include improvement to soil physical properties, maintenance of soil fertility, and disruption of pest and disease cycles (Chalk, 1998; Mandal et al., 2003; Stoddard et al., 2010; Adekiya et al., 2017). Nevertheless, there are some environmental risks, such as increases in N leaching or N_2_O emissions, associated with the use of faba bean in crop rotation; these risks can however be limited through appropriate rotation system design (Huth et al., 2010).

In summary, the main benefits of including faba bean in crop rotation systems are as follows: (1) reduced use of inorganic nitrogen fertilizers, (2) reduced CO_2_ emissions, (3) improved soil physical properties (i.e., bulk density, porosity, and water content at field capacity), (4) maintenance of soil fertility, and (5) higher yield and improved quality in subsequent crops.

Faba bean is usually employed as a break crop in cereal production. When rotated with cereals, it has proven to be beneficial in increasing the yield and seed protein content of successive cereal crops (Zou et al., 2015). In some Mediterranean countries, it is also incorporated into vegetable crop rotations, e.g., it can be utilized as a pre-crop of some summer crops, such as species in the Cucurbitaceae or Solanaceae families. Below are two examples of rotation sequences used in European cropping systems that include faba bean:

1. Faba bean (first year), cereal (second year), field or industrial crops (i.e., maize, cotton, tomato, sugar beet, oilseed rape; third year), and cereal (fourth year);
2. Faba bean – short period vegetable (first year), cereal (second year), field or industrial crops (i.e., maize, cotton, tomato, sugar beet, oilseed rape; third year), and cereal (fourth year).

### Soil Fertilization and Inoculation

Nitrogen fertilization is not generally required, but the application of “starter” nitrogen fertilization at a rate of 20 kg ha^-1^ seems to enhance the nodulation process in faba bean plants (Mohamed and Babiker, 2012). Furthermore, legume BNF is an energy intensive process that requires large amounts of phosphorus (P). Thus, P fertilization at a rate of 40 kg ha^-1^ can often enhance the nodulation process and N_2_ fixation, and increase yield (Bolland et al., 2000; Adak and Kibritci, 2016). Several other studies show that faba bean crops also respond to S and K fertilization (Sangakkara et al., 1996; Niewiadomska et al., 2015). Nevertheless, S or K fertilizers are rarely applied, because faba bean is cultivated as a low-input crop. Furthermore, micronutrient (e.g., zinc and boron) deficiencies are rare and can easily be corrected through foliar sprays.

Inoculating faba bean fields or seeds with *Rhizobium* is unnecessary in traditional cultivation areas. However, it is advisable to test their presence in the soil in areas where faba beans or other legumes have not been grown for several years. If absent, the crop can be inoculated with *Rhizobium leguminosarum* bv. *viciae* (Cubero, 2017). Dual inoculation with *Rhizobium* and arbuscular mycorrhizal fungi has been reported to be more effective than inoculation with *Rhizobium* alone in promoting faba bean growth, particularly in alkaline soils; this reflects the existence of synergistic relationships between the two inoculants (Abd-Alla et al., 2014).

### Irrigation

Faba bean usually grows without irrigation, with the exception of crops cultivated in very dry and hot climatic zones. Thus, production is highly dependent on the amount of and variation in rainfall during the growing season (Oweis et al., 2005). In semiarid regions, climate change can affect water use efficiency and growth in faba bean (Guoju et al., 2016), given its sensitivity to drought (Ghassemi-Golezani et al., 2009; Alghamdi et al., 2015). In the Mediterranean region and similar dry and hot climatic zones, faba bean production without irrigation may be possible if cultivation takes place during the cold season. Moreover, early sowing in autumn is considered an effective strategy for avoiding water stress during the seed filling stage (Loss and Siddique, 1997). Alternatively, faba bean crops can be irrigated at the seed filling stage in order to avoid penalties in yield during drought. Additionally, Knott (1999) reports that faba bean production is usually increased by irrigating spring crops during the flowering stage and early podding. Between 231 and 297 mm of water are required to produce 3–4.4 t ha^-1^ of faba bean dry biomass (Bryla et al., 2003). The development of drought-tolerant faba bean varieties is a key challenge in achieving increased and more stable production levels (Khan et al., 2010; Siddique et al., 2013). Several genotypes are considered tolerant to drought and can be exploited in breeding programs in order to develop drought-tolerant varieties (Ali, 2015). Recently, some varieties (e.g., CS20-DK and NC-58) have been evaluated as tolerant to water stress (Girma and Haile, 2014).

### Weed Control

Weed infestation is a major constraint in faba bean production, and can reduce yield by up to 50% (Frenda et al., 2013). Thus, early weed removal during the period between 25 and 75 days after sowing is necessary if a high yield is to be obtained (Tawaha and Turk, 2001). Similar to other winter pulse crops and cereals, the 12 main weeds that compete with faba bean in Europe are the broadleaved species *Anthemis arvensis* L., *Chenopodium album* L., *Papaver rhoeas* L., *Sinapis arvensis* L., *Fumaria officinalis* L., *Veronica* spp., *Lamium amplexicaule* L., *Cirsium arvense* (L.) Scop., and the grass species *Avena sterilis* L., *Phalaris* spp., *Lolium rigidum* Gaud., and *Alopecurus myosuroides* Huds. (Kalburtji and Mamolos, 2001; Karkanis et al., 2016a,b). Moreover, in many Mediterranean countries, such as Spain, faba bean can be parasitized by various broomrape species (*Orobanche* spp. and *Phelipanche* spp.) (Pérez-de-Luque et al., 2010); *Orobanche crenata* Frosk (bean broomrape) is the main species infesting faba bean in this area (Pérez-de-Luque et al., 2016).

Faba bean exhibits a superior ability to compete with weeds compared with other pulse crops, such as chickpea, due to its more vigorous early growth and greater plant height (Frenda et al., 2013). Nevertheless, the application of herbicides is a primary method in controlling weeds in conventional faba bean production. To our knowledge, the herbicides pendimethalin, clomazone, bentazon, quizalofop-*p*-ethyl, and propaquizafop are registered for use on this crop in the European Union. The first two are applied pre-emergence to control broadleaved and grass weeds; quizalofop-*p*-ethyl and propaquizafop are applied post-emergence to control grass weeds such as *Phalaris* spp. and *Lolium* spp., while bentazon is applied post-emergence to control broadleaved weeds. Crop rotation with spring crops can significantly reduce weed pressure, while allowing field application of herbicides that are not registered for use on faba bean (Karkanis et al., 2016b). Residual herbicides can damage faba bean planted in fields where chlorsulfuron (sulfonylureas) and aminopyralid (pyridine carboxylic acids) have previously been applied.

Currently, the development of resistant faba bean varieties would appear to be the most effective strategy for preventing broomrape infestation. In a recent study conducted in Egypt, Spain, and Tunisia by Rubiales et al. (2014), some accessions and the variety “Baraca” proved to be the most resistant to *O. crenata*. Several studies have also shown that late sowing and intercropping with cereals can reduce broomrape infection of faba bean (Pérez-de-Luque et al., 2004; Fernández-Aparicio et al., 2007; Abbes et al., 2010), while soil solarization is a non-chemical and effective method for controlling *O. crenata* and other weeds (Haidar and Sidahmed, 2000; Mauromicale et al., 2001).

### Disease and Insect Management

#### Diseases

Fungal diseases can severely damage faba bean crops, especially in wet weather conditions. Ascochyta blight, chocolate spot, and rust are the three main pathogens affecting faba bean crops globally (Torres et al., 2006; Stoddard et al., 2010). Ascochyta blight is caused by *Ascochyta fabae* Speg. (teleomorph *Didymella fabae* Jellis and Punithalingam), and is one of the most serious pathogens, causing up to 30% loss in yield (Davidson and Kimber, 2007; Omri Benyoussef et al., 2012; Ahmed et al., 2016). Although the application of fungicides, such as azocystrobin and chlorothalonil, considerably reduces ascochyta blight infection, integrated management practices (e.g., crop rotation, use of resistant varieties, and late sowing) are crucial to successful control (Davidson and Kimber, 2007; Stoddard et al., 2010; Ahmed et al., 2016). In a recent study, Rubiales et al. (2012) report that the faba bean accessions V-26, V-255, V-958, V-1020, V-1085, V-1117, and L-831818 showed good levels of resistance to *A. fabae*.

Chocolate spot is caused by the fungi *Botrytis fabae* Sard. and *Botrytis cinerea* Pers., while *Uromyces viciae-fabae* (Pers.) J. Schröt causes rust disease in faba bean (Emeran et al., 2011; Fernández-Aparicio et al., 2011; Abd El-Rahman and Mohamed, 2014). Rust and chocolate spot infection can cause yield losses of 22–42% and 36–68%, respectively (Sahile et al., 2010; Emeran et al., 2011). According to Emeran et al. (2011), foliar spraying with fungicides such as the triazoles (difenoconazole, epoxiconazole, or tebuconazole), dithiocarbamates (thiram, maneb, or mancozeb), and chlorothalonil was effective in controlling rust. In addition, procymidone is very effective against *B. fabae* (Marcellos et al., 1995), and chocolate spot severity in faba bean is reduced by frequent application of mancozeb (Sahile et al., 2008), intercropping with cereals such as barley, oat, triticale, and wheat (Sahile et al., 2008; Fernández-Aparicio et al., 2011), and low crop density and wide row spacing (Davidson et al., 2007). In a recent study, Mbazia et al. (2016) also observe that isolates of *Trichoderma viride*, *T. harzianum*, and *Bacillus subtilis* reduced chocolate spot severity in faba bean. A key option in the integrated management of *B. fabae* is the cultivation of resistant cultivars. According to Villegas-Fernández et al., 2012), the accessions 132-1, 135-1, 174-1, BPL 710, ILB 4726, and ILB 5284 exhibited a good level of resistance to *B. fabae* infection. Thus, these genotypes constitute an interesting genetic resource for future exploitation in breeding programs for developing chocolate spot-resistant cultivars.

Faba bean is also susceptible to viruses, with the principal sources of infection being faba bean necrotic yellows virus (FBNYV) and bean yellow mosaic virus (BYMV) (Ortiz et al., 2006; Shiying et al., 2007). Other diseases affecting faba bean crops are black root rot [*Fusarium solani* (Mart.) Sacc.], faba bean root rot (*Aphanomyces euteiches* Drechs.), powdery mildew (*Erysiphe pisi* var. *pisi*), and stem rot (*Sclerotinia trifoliorum* Erikss.) (Cook and Fox, 1992; Lithourgidis et al., 2003; van Leur et al., 2008; Habtegebriel and Boydom, 2016).

#### Insects

Several insects have the potential to infest faba bean plants. The black bean aphid (*Aphis fabae* Scop.) is a common pest (Hansen et al., 2008); aphids infest new leaves on faba bean plants (Nuessly et al., 2004). Foliar insecticide sprays (i.e., thiacloprid, fenvalerate) are very effective against these pests (Purhematy et al., 2013). Moreover, parasitoids play a significant role in the natural control of aphids (Boivin et al., 2012). *Lysiphlebus fabarum* Marshall (Hymenoptera) is a parasitoid of black bean aphid, and could prove useful as a biological control (Mahmoudi et al., 2010).

Other insects that infect faba bean crops are the pea leaf weevil (*Sitona lineatus* L.) and broad bean weevil (*Bruchus rufimanus* Boh.; Evenden et al., 2016; Seidenglanz and Huņady, 2016). *S. lineatus* adults feed on the foliage, while the larvae feed on faba bean and pea root nodules, affecting their ability to fix nitrogen (Cárcamo et al., 2015); treating seeds with thiamethoxam could be useful in controlling this insect (Cárcamo et al., 2012). Furthermore, storage pests, such as *B. rufimanus*, can cause significant yield losses in legumes; insecticides are however effective against them (Keneni et al., 2011).

## Genetic Material: Yield

Faba bean has a long history of cultivation. A broad gene pool has therefore been developed over several centuries, including local landraces, mass selections from landraces, open-pollinated populations, inbred lines, and cultivars (Duc et al., 2010). In addition, socioeconomic changes have led to decreases in cultivation and the disappearance of local genetic resources, with only small farms continuing to grow different landraces selected for their adaptation to local environmental conditions (Karaköy et al., 2014).

**FIGURE 2:**
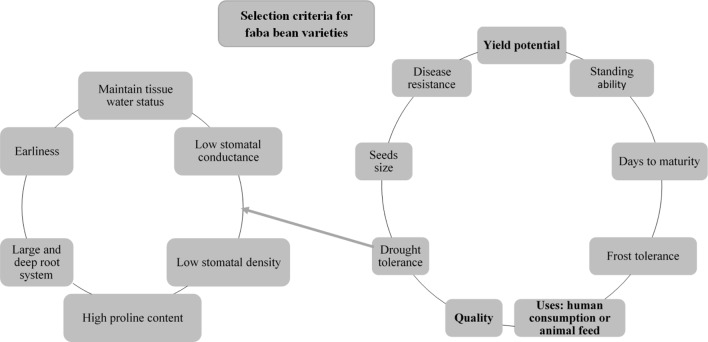
Main selection criteria for faba bean varieties (right-hand side); specific criteria for drought tolerance are indicated in detail (left-hand side).

Investment in legume breeding has been lower than for cereals (Fouad et al., 2013; Duc et al., 2015b) and, as a result, only a limited number of registered faba bean cultivars is available. For example, only 256 faba bean cultivars are currently registered for growing in Europe, and recorded at the EU database of registered plant varieties; for wheat (*Triticum aestivum*); however, there are 2415 registered cultivars (European Commission, 2017). “Aguadulce,” “Extra,” “Precose,” “Tundra,” “Fuego,” “Extra Violetto,” “Babylon,” and “Pyramid” are some commercial varieties cultivated in Europe under a wide range of agroclimatic conditions. Registered varieties feature a range of highly differing characteristics although genetic variation is limited (Fouad et al., 2013). Traits that should be targeted in selecting faba bean varieties include yield potential, quality, consistent performance, suitability for human consumption or the feed market, seed size, days to maturity, and standing ability (**Figure 2**), as well as resistance to disease and abiotic stress (see above sections).

The primary focus of legume breeding, including faba bean, is yield. However, in many regions, faba bean crops are subject to different conditions of biotic and abiotic stress and, consequently, yield is ultimately dependent on cultivar resilience to multiple stress conditions. Hence, breeding new cultivars with increased resilience to abiotic stresses, such as heat and salinity, continues to challenge breeders (Siddique et al., 2013; Nebiyu et al., 2016). Furthermore, winter hardiness is an important trait in screening for cultivars to be cultivated during the cold season (Link et al., 2010).

The genetic improvement of desired traits *via* breeding significantly depends on genetic variation in those traits. There is therefore an urgent need to collect and evaluate local genetic resources that can be used in well-designed breeding programs as donors of valuable features in the development of new improved varieties. In this sense, local landraces represent important sources for plant breeding, as they contain co-adapted genes that may prove valuable in future cultivation practices and in enhancing yield and quality (Harlan, 1975). The importance and wide variation of traits relating to morphology, agronomy, and quality have been previously investigated and demonstrated for local faba bean genetic resources of different origins: Turkey (Karaköy et al., 2014), the Mediterranean (Terzopoulos and Bebeli, 2008), Albania (Nasto et al., 2016), Palestine (Basheer-Salimia et al., 2014), and China (Zong et al., 2009). However, only a small proportion of other faba bean genetic resources has so far been evaluated. Thus, efforts to characterize the available resources should be intensified, and the collection of new local resources is crucial, because of the genetic erosion that is currently identified. Furthermore, breeding programs need to incorporate a more complex evaluation and integrated use of traits (Duc et al., 2015b). The abovementioned traits and others, such as root architecture (Zhao et al., 2018), shoot architecture, parameters related to stomatal function (Khan et al., 2010; Khazaei et al., 2013), and multiple disease resistance (Torres et al., 2006), are becoming increasingly important. It is apparent that any new variety should combine as many of the abovementioned characteristics as possible, in order to allow for a greater and more stable production of faba bean in specific agroecological zones.

The anticipated dry seed yield in faba bean crops ranges between 1.6 and 5.2 tons ha^-1^ (Argaw and Mnalku, 2017; Youseif et al., 2017), and the fresh pod yield ranges between 1.34 and 17.04 tons ha^-1^ (Baginsky et al., 2013; Etemadi et al., 2017). Faba bean yield components, such as the thousand seed weight, number of pods per plant, and number of seeds per pod, together with the duration of phenological stages and plant height, are correlated to grain yield (Sharifi, 2014; Bodner et al., 2018).

Yield stability and quality is a major objective of faba bean breeders (Alghamdi et al., 2012; Flores et al., 2013), since yield instability is a common problem encountered in cultivating this species and is considered a main cause of the decline in faba bean acreage (Suso et al., 1996; Flores et al., 2013). The stability of faba bean genotypes in different environmental conditions also needs to be examined (Temesgen et al., 2015). Several approaches can be applied in evaluating yield stability. Temesgen et al. (2015) demonstrate that different stability parameters have varying effects on yield performance, and recommend the application of several stability parameters, rather than only considering yield in different years. Faba bean genotypes exhibit a strong interaction with environmental conditions (Flores et al., 2013; Maalouf et al., 2015; Temesgen et al., 2015). In their study, Temesgen et al. (2015) report that the environment (E) accounted for 89% of yield variation, while the genotype (G) and the “G × E” interaction contributed 2 and 3%, respectively. The breeding of genotypes adapted to specific climatic zones is recommended in order to increase yield stability (Flores et al., 2013). G × E interactions are more common in faba bean than in most other crops; Bond (1987) reports that genotype × season interactions generally make a greater contribution than genotype × location.

## Harvest, Processing, Nutritional Value, and Use of Faba Bean

Faba bean crops cultivated for fresh seed consumption may be harvested either manually or mechanically once the pods are filled, but before they start to dry. Pods are harvested by hand two to three times during the harvesting period in crops cultivated in small areas for fresh consumption. When faba bean plants are cultivated for their dry seeds, they can be harvested using a conventional cereal combine harvester. Similar to other pulses, proper selection of the harvest stage is critical if seed loss is to be minimized (Karkanis et al., 2016a); seeds should be harvested when the moisture content is 14–15% (Jilani et al., 2012). In some countries (such as Canada), diquat is registered as a preharvest desiccant, and its application is a common practice among pulse growers (McNaughton et al., 2015), as this helps farmers to overcome problems caused by slow ripening and weeds during the harvest period.

Faba bean seeds also contain antinutrient compounds. Soaking, dehulling, boiling, pressure-cooking, autoclaving, and extrusion cooking are the main processing methods used to reduce the amounts of these compounds in faba bean seeds, in order to limit their adverse effects on human health (Luo and Xie, 2013; Patterson et al., 2017; Shi et al., 2017). Dehulling is efficient in eliminating the tannin and polyphenol content (Alonso et al., 2000), while soaking and autoclaving inactivate trypsin inhibitor activity (Luo and Xie, 2013; Shi et al., 2017).

The inclusion of plant-based proteins in human diets has a beneficial effect on human health (Moorthi et al., 2015). Faba bean protein content is reported to vary between 17.6 and 34.5% of seed dry matter, while acid detergent fiber (ADF) ranges between 10.1 and 13.7% (**Table 3**). Faba bean is also a valuable source of amino acids, being particularly rich in the essential amino acids arginine, lysine, and leucine, at up to 67 g kg^-1^ dry matter (Koivunen et al., 2016). As faba bean also provide macro-, micro-, and non-nutrient phytochemicals, it has been noted to have potential as a functional food. For example, Brauckmann and Latté (2010) report that faba bean seeds contain L-3,4-dihydroxyphenylalanine (L-DOPA), the precursor to the neurotransmitter catecholamine and a drug used to treat Parkinson’s disease.

**Table 3:** Nutritional value of faba bean dry seeds in comparison with two other important legumes widely cultivated in Europe.

Traits	Faba bean	Field pea	Lentil	References
Protein content (%)	17.6–34.5	19.9–27.6	25.8–28.6	Gharibzahedi et al., 2012; Duc et al., 2015a; Koivunen et al., 2016; Çalıcskantürk et al., 2017; Lepse et al., 2017; Li and Ganjyal, 2017; Bodner et al., 2018
Ash (%)	3.4–3.7	2.9–3.2	3.4–3.6	Gharibzahedi et al., 2012; Koivunen et al., 2016; Çalıcskantürk et al., 2017
ADF (%)	10.1–13.7	7.5–8.6	5.6–5.7	Gharibzahedi et al., 2012; Jezierny et al., 2017
NDF (%)	12.6–16.5	10.5–12.6	8.2–8.7	Gharibzahedi et al., 2012; Jezierny et al., 2017
Starch (%)	42.1–45.6	41.5–53.5	43.5–50.0	Jezierny et al., 2017; Li and Ganjyal, 2017
Minerals (mg kg^-1^)		
Fe	29.7–96.3	47.7–58.1	66–100	Gharibzahedi et al., 2012; Baloch et al., 2014; Ray et al., 2014
Zn	10.4–49.3	27.4–34.0	36.7–50.6	Gharibzahedi et al., 2012; Baloch et al., 2014; Ray et al., 2014
Mn	15.5–29.2	9–15.6	12.2–14.8	Gharibzahedi et al., 2012; Baloch et al., 2014; Ray et al., 2014
K	4500–19,300	9265–11,874	8802–10,240	Gharibzahedi et al., 2012; Baloch et al., 2014; Ray et al., 2014

*ADF, acid detergent fiber; NDF, neutral detergent fiber*.

Faba bean also contains antinutritional compounds such as saponins, lectins, tannins, vicine, convicine, and phytic acid (Hendawey and Younes, 2013; Multari et al., 2015). Tannins are known to reduce protein digestibility, while the absence of tannin in zero-tannin faba beans is controlled by either of the two genes zt-1 and zt-2 (Gutierrez et al., 2008; Woyengo and Nyachoti, 2012). The consumption of faba bean products containing high levels of vicine and convicine causes favism in humans, which is associated with glucose-6-phosphate dehydrogenase deficiency (Khamassi et al., 2013b).

Faba bean seed size is an important trait in determining market and consumption form. Large-seeded varieties (broad beans) are widely used for food, either as a fresh green vegetable or (dehulled) dry seeds. Varieties with small- to medium-size seeds are mostly used for animal feed (Crépon et al., 2010). Faba bean can also be used in the bakery industry (Belghith-Fendri et al., 2016); for example, a combination of faba bean and wheat flour improves the nutritional properties of bread (Coda et al., 2017). In Spain, small faba bean seeds (<12 mm) are currently highly accepted in the industry (Cubero, 2017). Small-seed genotypes are generally preferred by the frozen faba bean (Baginsky et al., 2013) and canning industries; the ability to use a microwave oven encourages the consumption of this legume, because seeds are much more easily cooked, and bags can be stored for up to 10 days at 5°C (Collado et al., 2017).

## Conclusion

Faba bean is important both as a pulse and a vegetable crop. The dry and fresh seeds or pods are recommended for their benefits to human nutrition as a dietary source of fiber and protein. Moreover, from an agronomical point of view, including faba bean in crop rotation systems improves soil, since this crop can fix atmospheric N_2_ to amounts that may exceed 200 kg N ha^-1^, and increases soil organic matter. Its inclusion in rotation systems therefore contributes to significant improvements in the sustainability of agricultural systems.

Fewer varieties of faba bean are recorded in comparison with other species in the European Union database. Production of this legume species is vulnerable to biotic and abiotic stresses, such as ascochyta blight, broomrape infestation, waterlogging, and drought. These constraints require there to be an urgent and increased focus on the development of new varieties that are resilient to these stresses. The new varieties should combine many of the above-mentioned characteristics, with the ultimate objective of achieving a high yield and high protein content.

## Author Contributions

AK, GN, ER, and DS developed the initial concept and outline, expanding the content, edited and revised the paper. DS, ER, and AB wrote the sections “Introduction,” “Irrigation,” and “Conclusions.” BR and GB wrote the sections: “Morphological description and botanical characterization” and “History, origin, and distribution.” LL wrote the section “Nutritional value and use of faba bean.” GN wrote the section “Faba bean adaptability to abiotic stress.” JF and MO wrote the section “Crop sowing and rotation.” IA wrote the section “Soil fertilization and inoculation.” AK wrote the sections “Weed control,” “Disease,” and “Insect management.” AKr, MO, LD, GN, and IV wrote the sections “Genetic material: yield, harvest, and processing of faba bean.”

## Conflict of Interest Statement

The authors declare that the research was conducted in the absence of any commercial or financial relationships that could be construed as a potential conflict of interest.
